# Artificial Intelligence, System Analysis and Simulation Modeling in Precise Prediction of 5-Year Survival of Esophageal Cancer Patients after Complete Esophagogastrectomies

**DOI:** 10.1186/1749-8090-10-S1-A153

**Published:** 2015-12-16

**Authors:** Oleg Kshivets

**Affiliations:** 1 Surgery Department, Kaluga Cancer Clinical Center, Kaluga, 248007, Russia

## Background/Introduction

Modern TNM-classification is based only on cancer characteristics and does not take into account at all the features of extremely complex alive supersystem - the patient's organism.

## Aims/Objectives

We examined factors in terms of precise prediction of 5-year survival (5YS) of esophageal cancer (EC) patients (ECP) (T1-4N0-2M0) after complete (R0) esophagogastrectomies (EG).

## Method

We analyzed data of 491 consecutive ECP (age = 56.2 ± 8.8 years; tumor size = 6.3 ± 3.4 cm) radically operated and monitored in 1975-2015 (m = 359, f = 132; EG Garlock = 280, EG Lewis = 211, combined EG with resection of pancreas, liver, diaphragm, aorta, VCS, colon transversum, lung, trachea, pericardium, splenectomy = 147; adenocarcinoma = 279, squamous = 202, mix = 10; T1 = 90, T2 = 112, T3 = 166, T4 = 123; N0 = 227, N1 = 69, N2 = 195; G1 = 136, G2 = 123, G3 = 232; early EC = 71, invasive = 420; only surgery = 377, adjuvant chemoimmunoradiotherapy-AT = 114: 5-FU+thymalin/taktivin+radiotherapy 45-50 Gy). Multivariate Cox modeling, clustering, SEPATH, Monte Carlo, bootstrap and neural networks computing were used to determine any significant dependence.

## Results

Overall life span was 1776.1 ± 2223.2 days and cumulative 5-year survival (5YS) reached 47.1%, 10 years - 40.3%, 20 years - 30%. 147 ECP lived more than 5 years, 79 - 10 years. 223 ECP died because of EC. Cox modeling displayed (Chi2 = 293.38, df = 18, p = 0.000) that 5YS of ECP significantly depended on: phase transition (PT) N0-N12 in terms of synergetics, cell ratio factors (CRF) (ratio between cancer cells and blood cells subpopulations), T, G, age, AT, localization, blood cells, prothrombin index, coagulation time, residual nitrogen (p = 0.000-0.014). Neural networks, genetic algorithm selection and bootstrap simulation revealed relationships between 5YS and PT N0--N12 (rank = 1), T, AT, G, prothrombin index, glucose, blood cells, localization, PT early-invasive EC, CRF. Correct prediction of 5YS was 100% by neural networks computing.

## Discussion/Conclusion

5YS of ECP after radical procedures significantly depended on: 1) PT "early-invasive cancer"; 2) PT N0--N12; 3) CRF; 4) blood cell circuit; 5) biochemical factors; 6) hemostasis system; 7) adjuvant chemotherapy; 8) tumor localization.

